# Effects of Organic Modification of Montmorillonite on the Properties of Hydroxypropyl Di-Starch Phosphate Films Prepared by Extrusion Blowing

**DOI:** 10.3390/ma11071064

**Published:** 2018-06-23

**Authors:** Yang Qin, Wentao Wang, Hui Zhang, Yangyong Dai, Hanxue Hou, Haizhou Dong

**Affiliations:** 1 Department of Food Science and Engineering, Shandong Agricultural University, 61 Daizong Street, Tai’an 271000, China; qyghostz@sdau.edu.cn (Y.Q.); wwtlxm@126.com (W.W.); zhanghui@sdau.edu.cn (H.Z.); dyyww@sdau.edu.cn (Y.D.); 2 Engineering and Technology Center for Grain Processing of Shandong Province, Tai’an 271000, China

**Keywords:** hydroxypropyl di-starch phosphate, montmorillonite, nanocomposite, extrusion blowing

## Abstract

The knowledge gained from starch-nanocomposite-film research has not been fully applied commercially because of the lack of appropriate industrial processing techniques for nanofillers and starch films. Three organically modified montmorillonites (OMMTs) were prepared using a semidry kneading method. The effects of the OMMTs on the structures and properties of starch nanocomposite films, prepared by extrusion blowing, were investigated. The X-ray diffraction (XRD) analysis results revealed that the OMMTs with various quaternary ammonium salts possessed differing layer structures and d-space values. The results of the XRD and Fourier-transform infrared spectroscopy (FT-IR) showed that the starch–OMMT interaction resulted in a structural change, namely the starch–OMMT films possessed a balanced exfoliated and intercalated nanostructure, while the starch–MMT film possessed an exfoliated nanostructure with non-intercalated montmorillonite (MMT). The results of the solid-state nuclear magnetic resonance (NMR) analysis suggested that the starch-OMMT nanocomposite possessed comparatively large quantities of single-helix structures and micro-ordered amorphous regions. The starch–OMMT films exhibited good tensile strength (TS) (maximum of 6.09 MPa) and water barrier properties (minimum of 3.48 × 10^−10^ g·m·m^−2^·s^−1^·Pa^−1^). This study indicates that the addition of OMMTs is a promising strategy to improve the properties of starch films.

## 1. Introduction 

Traditional plastic packaging materials such as polyethylene, polypropylene, and polyvinyl chloride, which are derived from petroleum sources, are not biodegradable and cause serious ecological problems [1]. To prevent pollution due to nondegradable plastics and improve environmental sustainability [2], many biodegradable polyesters [3] and natural macromolecules [4] have been used as substitutes for petrochemical materials in the preparation of biodegradable plastics. 

Starch is considered one of the most promising biodegradable substitutes for petrochemical plastics because of its abundance, low cost, renewability, and harmless degradation [5]. However, poor mechanical properties and high water sensitivity of starch films have limited their industrial application [6]. Many studies have been conducted to improve the performance of starch-based materials with various plasticizers. During the plasticizing process, the plasticizers form hydrogen bonds with starch, substituting for the strong interactions among the starch hydroxyl groups and causing plasticization. Glycerol is widely used as a plasticizer in starch-based materials due to low cost, nontoxicity, and a high boiling point [7,8,9]. Starch has been blended with polyesters [10] and cellulose [11]. However, the knowledge gained from research on starch films has not been fully utilized in their commercial application. 

Owing to its multilamellar structure, extremely large surface area, and intercalation properties, montmorillonite (MMT) has been used as an enhancer to improve the properties of bio-polyesters or starch plastics [12]. In addition to its structural advantages, MMT can be easily modified using various organic modifiers to develop new interfacial features and large lamellar spaces [13]. As preferred modifiers, quaternary ammonium salts exhibit superior performances to other surfactants on intercalated or exfoliated MMT layers [14]. Through the use of quaternary ammonium salts with various alkyl chain lengths and chemical groups, organically modified montmorillonites (OMMTs), with various layer structures and d-spacings, have previously been prepared in aqueous solutions and used to enhance starch films [15]. The mechanical and barrier properties of various types of starch films have been greatly enhanced through the incorporation of MMTs and OMMTs via solution casting [16]. However, considering the preparation of starch films, the solution-casting method is considered more suitable for laboratory research purposes because it is discontinuous and inefficient. Few researchers have studied the effects of MMTs on starch films produced by extrusion blowing [17,18], which is considered to be an effective method for the continuous production of starch film [19]. Therefore, the effects of OMMTs, namely their layer structure and dispersion, on starch films produced via extrusion blowing should be further studied to improve the mechanical and barrier properties of starch nanocomposites. In addition, the OMMTs used in starch nanocomposites are usually prepared using the solution method [15], which results in large quantities of waste water and low yields of OMMTs. Hence, to meet commercial requirements, a new process should be developed for the manufacture of OMMTs. 

In this work, OMMTs with various layer structures were prepared. This was achieved, using a semidry kneading method, by modifying MMT-Na^+^ using three quaternary ammonium salts with various alkyl-chain lengths. The obtained OMMTs were then used to enhance starch films prepared by extrusion blowing. The multiple structural changes (owed to the introduction of the OMMTs occurring in the starch films) were investigated, in particular, the changes in the short-range- and micro-ordered regions. In addition, the changes in the mechanical properties and water vapor permeability (WVP) were investigated. The objective of this study is to reveal the mechanisms associated with various OMMTs, produced by a semidry kneading process, regarding their effects on the nanostructures and performances of starch films produced by extrusion blowing. 

## 2. Materials and Methods 

### 2.1. Materials

Hydroxypropyl di-starch phosphate (HPDSP) was purchased from Hangzhou Starpro Co., Ltd. (Hangzhou, China). MMT-Na^+^, with a cation exchange capacity (CEC) and d-spacing of 139.4 mmol/100 g and 1.47 nm, respectively, was purchased from Shouguang Zhonglian Fine Montmorillonite Co., Ltd. (Weifang, China). The surfactants that were used to modify MMT-Na^+^ were purchased from Shandong Long Chain Chemical Co., Ltd. (Binzhou, China). Anhydrous ethanol and glycerol were purchased from Tianjin Kaitong Chemical Reagent Co., Ltd. (Tianjin, China).

### 2.2. Preparation of OMMTs 

MMT-Na^+^ was modified using three quaternary ammonium salts, via a semidry kneading method. Quaternary ammonium salt (750 g) was dissolved in anhydrous ethanol (400 g) in a water bath at 80 °C. Then, the surfactant solution was mixed with the MMT-Na^+^ (3 kg) that had been preheated at 80 °C in a kneading machine. The mud-like blend was kneaded at 80 °C for 60 min in a sealed kneading machine. This was then continued for another 30 min using an open kneading machine at the same temperature. Subsequently, the OMMT was washed in hot aqueous ethanol and dried in an oven for 12 h. Finally, it was ground and sifted through a 200-mesh sieve. The OMMTs containing modifiers 1231, 1631, and 1831 were denoted as 1231-MMT, 1631-MMT, and 1831-MMT, respectively. The structures of the quaternary ammonium salts, as well as the CEC values and d-spacings of the OMMTs determined according to the method of Gao et al. [20], are shown in Table 1. 

### 2.3. Preparation of HPDSP Nanocomposite Films 

HPDSP and 10% (*w*/*w*) MMT-Na^+^ or OMMT were blended in an SHR50A mixer (Hongji Machinery Co., Ltd, Zhangjiagang, China) for 5 min at 500 rpm. Then, 30% (based on the starch mass, *w*/*w*) glycerol was added, and the mixture was blended for 10 min at 1500 rpm. The samples containing MMT-Na^+^, 1231-MMT, 1631-MMT, and 1831-MMT were denoted as HPDSP-Na, HPDSP-1231, HPDSP-1631, and HPDSP-1831, respectively. The mixtures were sealed in polyethylene bags for 24 h at room temperature to equilibrate the components. 

The mixtures were compounded using a laboratory-scale twin-screw extruder (Mengniu Plastic Machinery Co., Ltd., Laiwu, China), with a screw diameter of 35 mm and screw length of 30 D; temperatures of 80 °C and 120 °C were used in zones I and II of the barrel, respectively, and a screw speed of 20 rpm was employed. 

The HPDSP nanocomposite films were blown using a single-screw extruder with a screw diameter of 35 mm, screw length of 25 D, screw compression ratio of 3:1, and five individually controlled temperature zones. The extruder was equipped with a conventional temperature-controlled film-blowing die with a diameter of 60 mm and a film-blowing tower with a calendering nip and takeoff rolls (Mengniu Plastic Machinery Co., Ltd., Laiwu, China). The temperatures between the feed inlet and the die were 80 °C, 120 °C, 130 °C, 135 °C, and 125 °C. The ratio of the diameter of the blown bubble to that of the die (blow-up ratio) was carefully adjusted to 3:1. At the die exit, the ratio of the take-up velocity to the film velocity (take-up ratio) was carefully adjusted to 4:1. Prior to testing, all the films were stored for at least seven days within a chamber maintained at a constant temperature of 23 °C and relative humidity (RH) of 53% (HWS-60, Shanghai Jinghong Laboratory Equipment Co., Ltd., Shanghai, China).

### 2.4. Characterization 

#### 2.4.1. Thickness 

The thickness of the HPDSP nanocomposite films was determined using 211-101F micrometers (Guanglu Measuring Instrument Co., Ltd., Guangxi, China). Each film was tested randomly for six times and the thickness of the film was obtained by averaging the results of the six replicates. 

#### 2.4.2. Mechanical Properties 

The mechanical properties of the HPDSP nanocomposite films were determined, according to the ASTM-D882-02 standard test method (2002), using a TA-XT2i texture analyzer (Stable Micro System Company, Godalming, UK). Each film was cut into five strips, each with a length and diameter of 90 and 15 mm, respectively. The initial distance between the grips was 50 mm, and the test speed was 60 mm/min. The tensile strength (TS, MPa) and elongation at break (EAB, %) values of the films were obtained by averaging the results of the six replicates. 

#### 2.4.3. Water Vapor Permeability (WVP) 

The WVP was determined using a PERMETMW3/030 water vapor permeability tester (Labthink Instruments Co. Ltd., Jinan, China). The films were cut into round specimens (80 mm in diameter) using a special sampler. The WVP was determined at a temperature and RH of 38.0 °C and 90%, respectively, using a preheating time of 4 h and weighing interval of 120 min. The WVP of each sample was obtained by averaging the results of the three separate tests. 

#### 2.4.4. X-ray Diffraction (XRD) 

X-ray diffraction (XRD) was conducted to analyze the MMT-Na^+^, OMMTs, and HPDSP nanocomposite films. This was performed using a D8 Advance X-ray diffractometer (Bruker-AXS, Karlsruhe, Germany) equipped with a copper target, λ = 0.15406 nm; the radiation parameters were 40 kV and 30 mA, and a slit of 2 mm was used. In the case of the MMT-Na^+^ and OMMT films, XRD patterns were acquired over the 2θ range of 1 to 10°, while a range of 1–30° was used for the HPDSP nanocomposite films. A step size and step rate of 0.02° and 0.5 s were used, respectively. 

#### 2.4.5. Attenuated Total Reflectance-Fourier-Transform Infrared (ATR-FTIR) Analysis 

Fourier-transform infrared (FT-IR) analysis was performed on the HPDSP nanocomposite films, using a Nicolet iS 5 FT-IR analyzers with an attenuated total reflectance (ATR) detector (Thermo Fisher Scientific, Waltham, MA, USA). The number of accumulated scans and the scanning rate were 32 and 4 cm^−1^, respectively. All spectra were baseline corrected over the range of 1200–900 cm^−1^. The line shape was assumed to be Lorentzian, with a resolution enhancement factor and half-width of 1.9 and 19 cm^−1^, respectively. The infrared absorbance values at 1047 and 1022 cm^−1^ were extracted from the spectra following baseline correction and normalization of the second derivative spectra using OMNIC software. Then, the ratio of the absorbance at 1047/1022cm^−1^ was determined. 

#### 2.4.6. Solid-State Nuclear Magnetic Resonance (NMR) 

Solid-state nuclear magnetic resonance (^13^C NMR) was performed using a Bruker Avance III 400 MHz (^1^H/proton resonance) Wide Bore (Bruker-AXS, Karlsruhe, Germany) equipped with a 4-mm broadband double-resonance CP/MAS probe. Each film sample (500 mg) was placed into the rotor and introduced to the center of a magnetic field. The spectra were decomposed using PeakFit software version 4.0 (Systat Software Inc, San Jose, CA, USA). 

#### 2.4.7. Statistical Analysis 

The data in the tables and figures are represented by the mean values and standard deviations obtained. Any significant differences in the group means, at the 95% significance level, were tested using analysis of variance (ANOVA). This was followed by post-hoc Duncan’s multiple range tests using SPSS software 21 (IBM Co., New York, NY, USA). 

## 3. Results and Discussion 

### 3.1. Layer Structures of the Organic Montmorillonites (OMMTs) 

**Figure 1:**
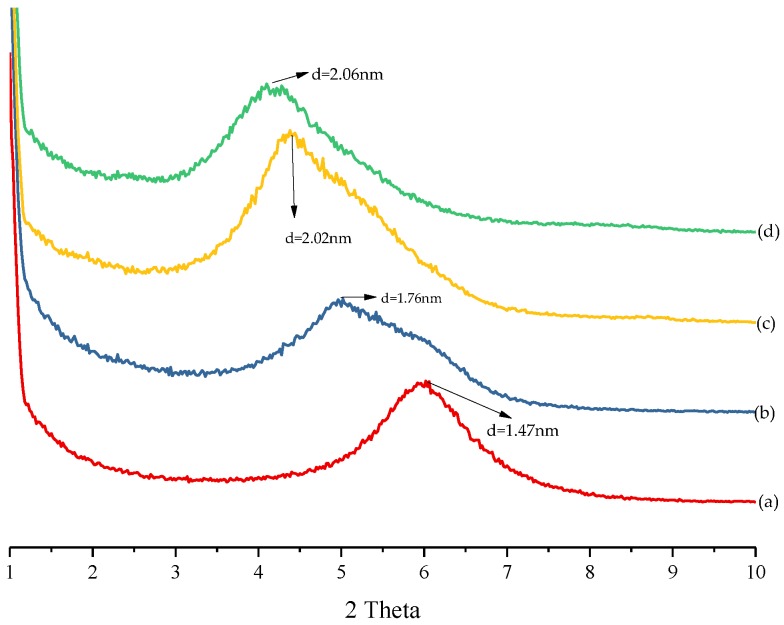
X-ray diffraction (XRD) patterns of (a) MMT-Na^+^; (b) 1231-MMT; (c) 1631-MMT; and (d) 1831-MMT.

The XRD patterns of MMT-Na^+^ and OMMTs modified by the various quaternary ammonium salts are shown in Figure 1. The shape of the characteristic peak reflects the intercalated or exfoliated state of the layers, and the peak position indicates the d-spacing of the lamellae, according to Bragg’s Law [21]. The characteristic peak of MMT-Na^+^ can be observed at 2θ = 5.98°, which is equivalent to a basal spacing of 1.47 nm. In the case of the OMMTs modified with quaternary ammonium salts, the characteristic peaks shift to lower angles. This indicates that surfactants 1231, 1631, and 1831 were intercalated into the MMT layers via the semidry kneading method. This resulted in the expansion of the d-spacings from 1.47 nm (MMT-Na^+^) to 1.76, 2.02, and 2.06 nm, respectively (Figure 1). These results indicate that the d-spacings of the OMMTs are positively related to the length of the alkyl chain of the modifier (R^2^ = 0.978, *p* = 0.05). By comparing the intensities and shapes of the peaks, it can be concluded that amongst the OMMTs, 1631-MMT had the most ordered layer structure, and 1231-MMT had the most disordered structure [22]. 

### 3.2. Layer Dispersion of the Various HPDSP Nanocomposite Films 

**Figure 2:**
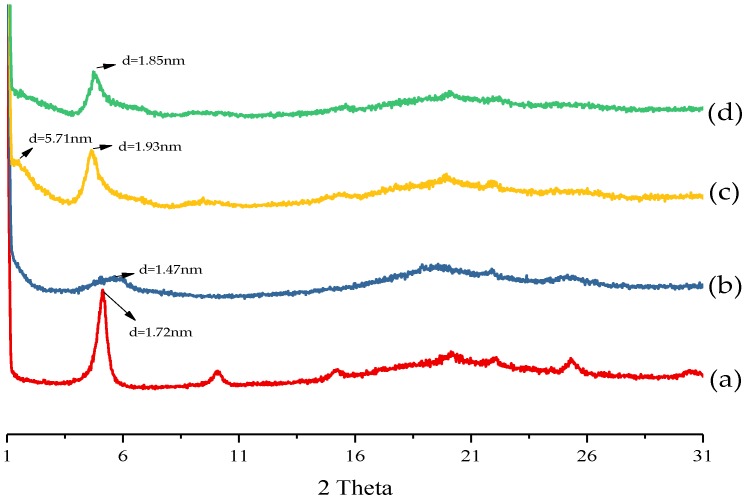
XRD patterns of (a) HPDSP-Na; (b) HPDSP-1231; (c) HPDSP-1631; and (d) HPDSP-1831.

The layer dispersion of MMT-Na^+^ and OMMTs within the HPDSP matrix was determined by measuring the characteristic peaks within the XRD patterns (Figure 2); these were then compared with those of the layer structures of MMT-Na^+^ and OMMTs (Figure 1). In the case of the HPDSP-Na film, the d-spacing of MMT-Na^+^ increased from 1.47 to 1.72 nm, indicating that a few starch chains were intercalated into the layers. Compared with that of MMT-Na^+^, shown in Figure 1, the characteristic peak of HPDSP-Na (Figure 2) became sharper. This suggests that the weak layers of MMT were exfoliated by starch molecules, and that the MMT-Na^+^ layers remained ordered in the HPDSP matrix [23]. Following the formation of the HPDSP-1231 nanocomposite, the d-spacing of the 1231-MMT decreased from 1.76 to 1.47 nm. This may be because the starch molecules primarily exfoliated the modified parts of MMT, but generally did not become intercalated into the unmodified parts. In the case of the HPDSP-1831 film, the starch molecules also exfoliated the 1831-MMT layers, which broadened the characteristic peak. However, some starch chains entered the MMT galleries, which increased the d-spacing to 1.85 nm. However, in the case of the HPDSP-1631 film, a new peak appeared at 2θ = 1.5°, which was not observed in the case of the other nanocomposites (Figure 2). This suggests that the starch molecules became further intercalated into the 1631-MMT layers, forming an orderly, weak composite structure [19]. 

### 3.3. Thickness of the HPDSP Nanocomposite Films

Thickness of the HPDSP nanocomposite films were showed in Table 2. The thickness of the HPDSP films with OMMTs were significantly thinner than the one of HPDSP-Na film at the same blow up ratio and take-up ratio.

### 3.4. Mechanical Properties of the HPDSP Nanocomposite Films 

**Figure 3:**
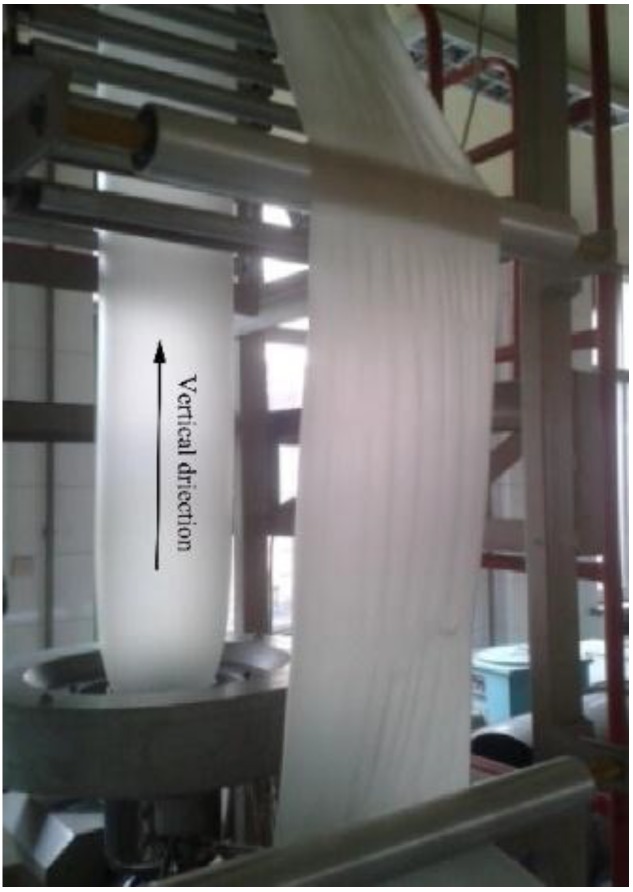
Continuous and stable blowing of HPDSP-OMMT film.

During extrusion blowing, a pulling force exists, which moves the starch film vertically. A tensile force also exists, which causes the film to expand. The TS and EAB values were measured in two directions, namely the vertical direction, which is the direction of the pulling force (Figure 3), and the horizontal direction, which is maintained at an angle of 90° to the pulling force. 

**Figure 4:**
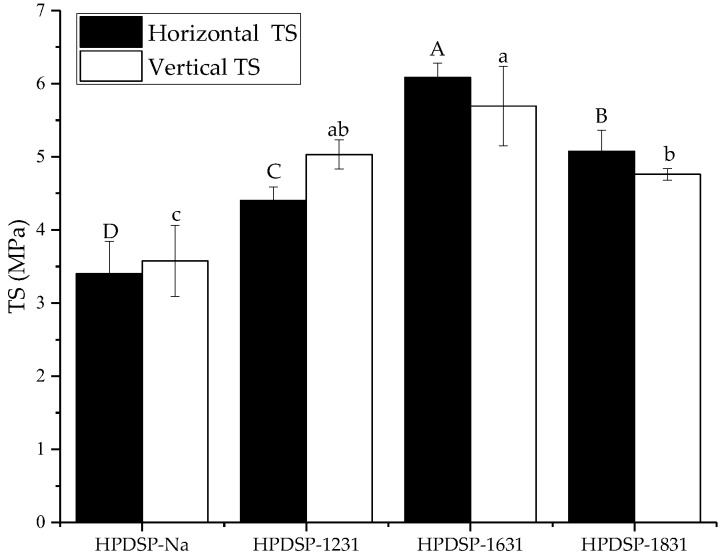
Horizontal and vertical tensile strength (TS) values of the HPDSP-Na and HPDSP-OMMT films. Results are quoted as means ± SD (standard deviation) of sextuple determinations. A–D and a–d: Different letters indicate significant differences among formulations (*p* < 0.05).

The TS values of the starch films with various OMMTs are shown in Figure 4. The TS value of the HPDSP-Na film was 3.40 MPa in the horizontal direction and 3.57 MPa in the vertical direction. When the starch films were enhanced with OMMTs, there were significant increases in the TS values in both the directions. The HPDSP-1631 film had the greatest TS values, namely 6.08 and 5.69 MPa in the horizontal and vertical directions, respectively. This result indicates that HPDSP and 1631-MMT form a homogeneous intercalated structure, which is assumed to facilitate stress transfer and improve the TS value [17]. 

**Figure 5:**
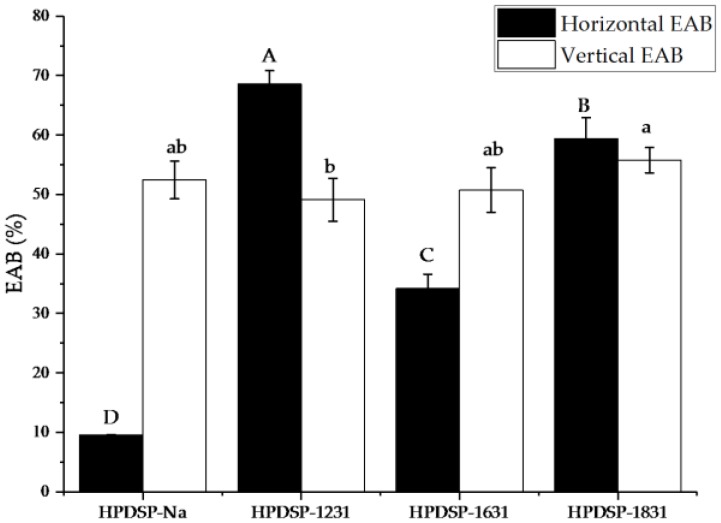
Horizontal and vertical elongation at break (EAB) values of the HPDSP-Na and HPDSP-OMMT films. Results are quoted as means ± SD (standard deviation) of sextuple determinations. A–D and a–d: Different letters indicate significant differences among formulations (*p* < 0.05).

The EAB values determined for the HPDSP-Na and HPDSP-OMMT films are shown in Figure 5. In the case of the HPDSP-Na film, the EAB values determined in the horizontal and vertical directions were 9.5% and 52.4%, respectively. The huge difference between these EAB values indicates that a molecular orientation effect exists, which is caused by the extrusion blowing process [24]. The addition of 1231-MMT, 1631-MMT, and 1831-MMT significantly increased the EAB values in the vertical direction to 68.6%, 34.1%, and 59.3%, respectively, while the HPDSP-1231 film possessed the best ductility. The OMMT was also effective in reducing the difference between the EAB values measured in the two directions (Figure 5). These results are consistent with the conclusions of other studies, which suggest that starch nanocomposites with OMMTs have relatively low EAB values and relatively high TS values [25]. 

### 3.5. Water Vapor Permeability (WVP) of the HPDSP Nanocomposite Films 

**Figure 6:**
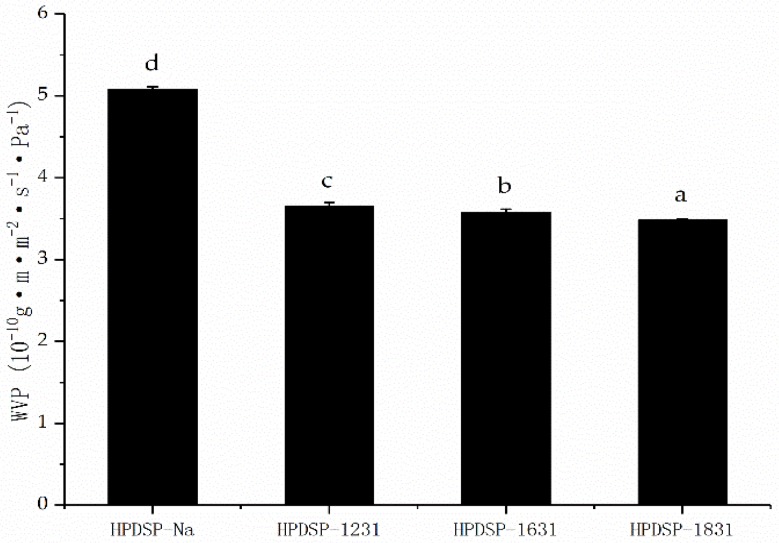
Water vapor permeability (WVP) of the HPDSP nanocomposite films. Results are quoted as means ± SD (standard deviation) of triplicate determinations. a–d: Different letters indicate significant differences among formulations (*p* < 0.05).

The WVP of the HPDSP-Na and HPDSP-OMMT films are shown in Figure 6. The addition of the OMMTs resulted in a significant decrease in the WVP of the starch films. The WVP decreased from 5.08 × 10^−10^ to 3.65 × 10^−10^, 3.58 × 10^−10^, and 3.48 × 10^−10^ g·m·m^−2^·s^−1^·Pa^−1^ in the cases of the HPDSP-1231, HPDSP-1631, and HPDSP-1831 films, respectively. The decrease in the WVP could be owed to the presence of a hydrophobic layer of OMMTs, which covered the starch molecules in the films and hindered the absorption of moisture [24]. In general, the transmission of water vapor through a film depends on both the solubility and diffusivity of the water molecules within the film matrix. When the HPDSP-OMMT nanocomposite structures were formed, the impermeable MMT layers prevented water diffusion, thereby increasing the effective pathway length of the water molecules traversing the film matrix [23]. Even though the 1231-MMT had a less hydrophobic surface than the other two OMMTs, the HPDSP-1231 film had a relatively low WVP value owing to the exfoliated 1231-MMT layers. In addition, the WVP value of the HPDSP-1631 film was slightly greater than that of the HPDSP-1831, although the hydrophobicity of the two modifiers was similar. This suggests that the exfoliated layers efficiently increase the length of the tortuous path of the water molecules.

### 3.6. Intermolecular Interactions that Occur within HPDSP Nanocomposite Films 

**Figure 7:**
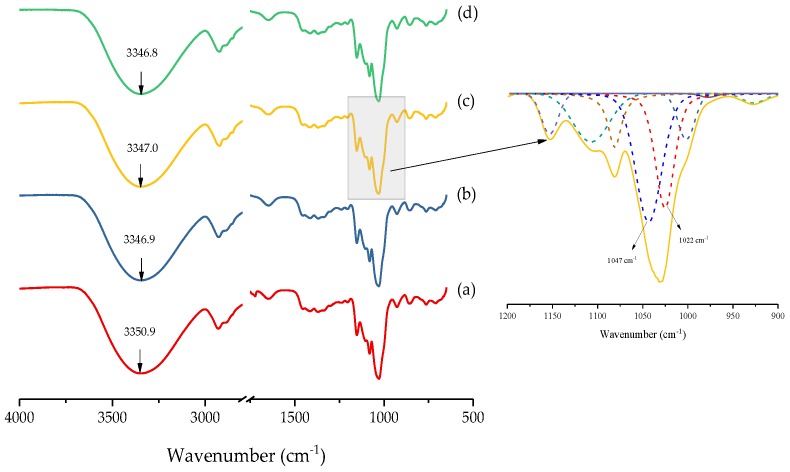
Fourier Transform Infrared Spectroscopy (FT-IR) spectra of (a) HPDSP-Na; (b) HPDSP-1231; (c) HPDSP-1631; and (d) HPDSP-1831 and a magnified view of the peaks between 1200 to 900 cm^−1^ from HPDSP-1631 spectrum (on the right).

FT-IR analysis can be used to effectively study the interactions that occur between the starch molecules and MMT layers. Figure 7 shows the FT-IR spectra of the HPDSP-Na and HPDSP-OMMT films. In the case of the HPDSP-Na film, a peak, owing to -OH stretching, appeared at 3350.9 cm^−1^. In the cases of all the HPDSP-OMMT films, this peak shifted to 3346.9 cm^−1^. According to the harmonic oscillator model, the IR peak frequency of -OH decreases as the number of molecular interactions increases [26]. Thus, this red shift demonstrates that the OMMT disrupts the inter- and intramolecular hydrogen bonds that exist between the starch granules and exposes the hydroxyl groups. This allows the formation of new hydrogen bonds between the starch molecules and OMMT layers [27]. 

With regard to starch, the FT-IR absorbance bands at 1047 and 1022 cm^−1^ are associated with ordered and amorphous short-range structures, respectively, and the 1047/1022 ratio is used to measure the change in the ratio of the amount of ordered starch to the amount of amorphous starch [28]. The 1047/1022 ratios of the HPDSP-OMMT nanocomposites were greater than that of the HPDSP-Na nanocomposite (Table 2). A high 1047/1022 ratio was determined for the HPDSP-1631 film. This could be because the 1631-MMT within the HPDSP-1631 film provided sufficient space between the intercalated layers for the formation of local, micro-ordered starch structures. This consequently enhanced the TS of the film. In the case of the other two starch nanocomposites, the exfoliated MMT layers effectively disrupted the hydrogen bonds, which are essential for the formation of disordered structures [29]. 

### 3.7. Short-Range Molecular Conformation of the HPDSP Nanocomposite Films

**Figure 8:**
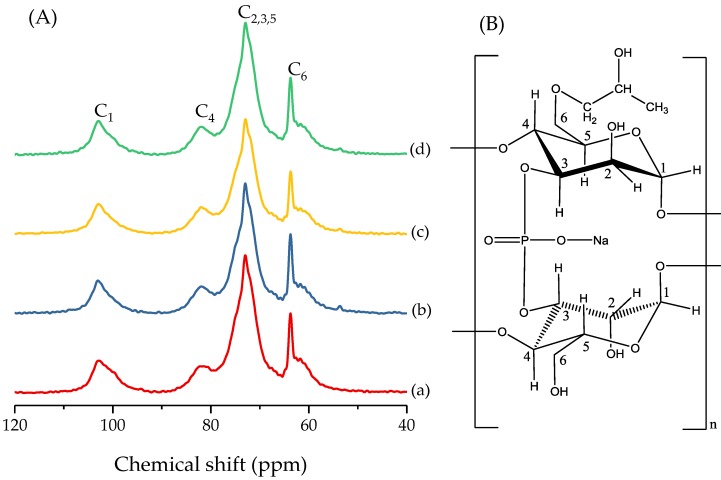
(**A**) ^13^C nuclear magnetic resonance (NMR) spectra of (a) HPDSP-Na; (b) HPDSP-1231; (c) HPDSP-1631; (d) HPDSP-1831; and (**B**) the HPDSP structure.

The ^13^C NMR spectra provide information on the changes that occur with regard to the short-range molecular organization of starch materials such as those associated with amorphous single and double helices [30]. As shown in the ^13^C NMR spectra of the starch nanocomposite film, four main peaks can be observed, which correspond to various glucose carbons (Figure 8A). The peaks C_1_ (100–103 ppm), C_4_ (81.4 ppm), C_2,3,5_ (72.2 ppm), and C_6_ (61.9 ppm) correspond to the carbon atoms within the HPDSP molecules, as shown in Figure 8B. 

The ordered spectrum at the C_1_ peak (100–103 ppm) can be resolved into the double-helix (series of peaks near 100 ppm) and single-helix (103 ppm) components of the starch molecule [31]. Considering the C_1_ peaks in the spectra of the HPDSP-Na film, a broad peak was observed near 103 ppm, and a series of shoulder peaks were observed near 100 ppm. These observations suggest that the starch molecules of the nanocomposites primarily consist of a single-helix V-type structure with a number of double helices. Following the addition of OMMT to the starch film, the area of the peak at 103 ppm increased (Table 3), and the shoulder peak at 100 ppm disappeared (Figure 8A). This indicates that the distribution of the OMMTs within the HPDSP matrix was superior to that of MMT-Na^+^. This promoted the double-helix structure to transform into a single-helix structure. A sharper C_1_ peak was observed in the spectra of the HPDSP-1631 film, which suggests that the 1631-MMT contributes to homogeneous distribution by interrupting inter- and intramolecular hydrogen bonds [23]. In addition, these results support the conclusions of previous studies, which suggest that the incorporation of homogeneous nanocomposites improves the mechanical properties of starch films [25].

**Figure 9:**
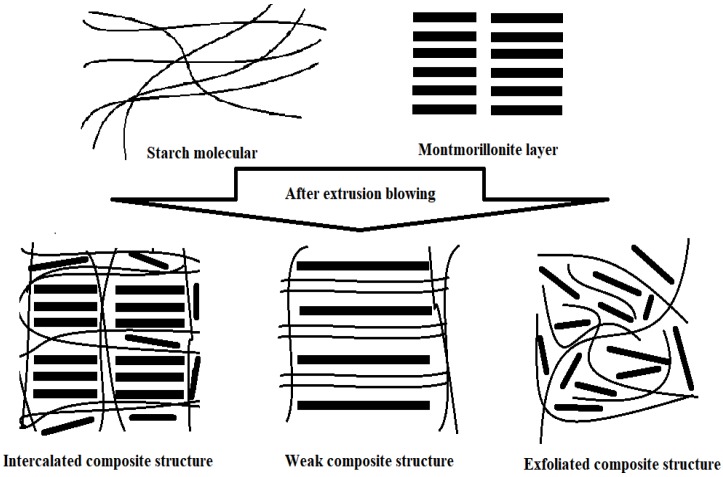
Schematic of composite structure of HPDSP with montmorillonite (MMT)

The C_4_ peak is considered characteristic of the amorphous glassy phase of starch [32]. When various OMMTs were added, there was a change in the C_4_ peak area. This suggests that further amorphization of the starch film occurred. The total change in the short-order structure of the starch film, prepared by extrusion blowing and with various OMMTs, was calculated using Liu’s method [33] (Table 3). In general, a significant total change in the short-order structure indicates further formation of amorphous regions within the starch film. Therefore, it was concluded that the HPDSP-1631 film had the largest amorphous region. However, this seems to contradict the results of FT-IR analysis, which indicate that the HPDSP-1631 film has the most ordered microstructure [29]. One reasonable explanation is that the amorphous structure of the HPDSP-1631 film was arranged into a micro-ordered structure via the formation of an orderly, weak composite structure [19], as indicated by the peak at 2θ = 1.5° within the HPDSP-1631 XRD pattern (Figure 3). A schematic of composite structures of HPDSP with various MMT was given to explain the weak composite structure (Figure 9). The weak composite structure in HPDSP-1631 film was a structure between intercalated and exfoliated structure, in which the starch molecular deeply intercalated into the layers but no exfoliated structure was formed; and the narrow space between layers forced the amorphous starch molecular line up orderly. This result validates the explanation regarding the mechanical properties of the HPDSP-MMT films, which suggests that the ordered arrangement of the starch molecules effectively resulted in an increase in the TS value but a decrease in the EAB value.

## 4. Conclusions

The effects of OMMTs, prepared by a semidry kneading process, on the properties of starch films, formed by extrusion blowing, were investigated. Following the addition of the OMMTs, the starch films exhibited higher TS values and improved EAB values. This was owing to the formation of a greater quantity of micro-ordered regions with single-helix structures, which prevented changes in the molecular orientation. Compared with the results obtained for the film containing MMT-Na^+^, the hydrophobic surface and exfoliated layers of the OMMTs enhanced the water vapor barrier. Various OMMTs influenced the proprieties of the starch film by changing the balance between the exfoliated and intercalated starch-MMT layer systems and the short-range structures of the amorphous and crystalline regions. The starch film with 1631-MMT had more single helix structures and micro-ordered amorphous regions, making it more suitable than 1231-MMT and 1831-MMT for enhancing HPDSP films prepared by extrusion blowing. The HPDSP nanocomposite film is a promising material for food inner packaging due to its good tensile strength and barrier properties. 

## Figures and Tables

**Table 1:** Characteristics of the three quaternary ammonium salts and organic montmorillonites (OMMTs).

Modifier Name (Short Name)	Chemical Structure of the Modifier	OMMTs	CEC (mmol/100 g)	d-Spacing (nm)
Dodecyl trimethyl ammonium chloride(1231)	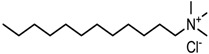	1231-MMT	96.4	1.76
Hexadecyl trimethyl ammonium chloride(1631)	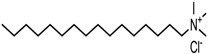	1631-MMT	89.2	2.02
Octadecyl trimethyl ammonium chloride(1831)	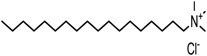	1831-MMT	87.3	2.06

**Table 2:** Thickness and ratios of 1047/1022 for HPDSP nanocomposite films.

Sample	HPDSP-Na	HPDSP-1231	HPDSP-1631	HPDSP-1831
Thickness/mm	0.452 ± 0.055 a	0.189 ± 0.003 b	0.146 ± 0.002 b	0.189 ± 0.002 b
Ratio of 1047/1022	0.77	0.774	0.800	0.776

Results are quoted as means ± SD (standard deviation) of triplicate determinations. a–d: Different letters indicate significant differences among formulations (*p* < 0.05).

**Table 3:** Positions of the C_1_ and C_4_ peaks of the HPDSP nanocomposite films and their respective areas.

Sample	C_1_						C_4_		Total Change/%
	Center/ppm	Area 1/%	Center/ppm	Area 2/%	Center/ppm	Area 3/%	Center/ppm	Area 4/%	
HPDSP-Na	102.90	13.04	100.95	1.55	99.50	3.18	81.30	6.16	0.00
HPDSP-1231	103.02	17.19	101.38	0.54	100.00	2.80	81.34	7.86	2.13
HPDSP-1631	102.93	13.15	100.84	1.27	100.84	1.18	81.36	11.27	6.24
HPDSP-1831	103.06	13.23	101.68	1.06	101.68	2.97	81.33	8.30	3.27
